# Granulomatosis With Polyangiitis in a Patient on Programmed Death-1 Inhibitor for Advanced Non-small-cell Lung Cancer

**DOI:** 10.3389/fonc.2019.00478

**Published:** 2019-06-06

**Authors:** Anne Sibille, Romain Alfieri, Olivier Malaise, Nancy Detrembleur, Michelle Pirotte, Renaud Louis, Bernard Duysinx

**Affiliations:** ^1^Department of Pulmonology, University Hospital of Liège, Liège, Belgium; ^2^Department of Internal Medicine, University Hospital of Liège, Liège, Belgium; ^3^Department of Rhumatology, University Hospital of Liège, Liège, Belgium; ^4^Department of Pathology, University Hospital of Liège, Liège, Belgium

**Keywords:** immune checkpoint inhibitor, non-small-cell lung cancer, granulomatosis with polyangiitis, immune-related adverse events, anti-PD-1 antibody

## Abstract

**Objectives:** To contribute to a precise and thorough knowledge of immune-related adverse events (irAE) induced by immune checkpoint inhibitors (ICI) and to emphasize the importance of this specific form of toxicity in terms of potential predictive value and long-term effects.

**Materials and Methods:** We report the first case of granulomatosis with polyangiitis (GPA) in a patient treated with an anti-Programmed Death protein-1 (PD-1) antibody for advanced non-small-cell lung cancer (NSCLC).

**Results:** After a single dose of this drug the patient showed severe myositis associated with a high anti-PR3 anti-neutrophil cytoplasmic antibody titer. Discontinuation of the anti-PD-1 and introduction of corticoids led to a remission of the irAE. Regarding tumor a partial response was noted. A year later a neutrophilic, sterile pleural exudate and cutaneous lesions appeared with the pathological findings of neutrophilic vasculitis. Retreatment with corticoids induced a new remission of symptoms. It remains unclear whether GPA was preexisting and clinically silent but revealed by the use of ICI or primarily induced by this treatment. Conclusions: irAE are rare when anti-PD-1 antibodies are used in monotherapy. They present with a distinct clinical picture and temporal course and require specific treatment. Patients with irAE usually have a favorable oncological outcome.

## Introduction

Immune-checkpoint inhibitors (ICI) have become widely used in advanced non-small-cell lung cancer (NSCLC). Classically, patients with preexisting autoimmune disorder (PAID) have been excluded from clinical trials using ICI. Real-life experience, however, shows that physicians sometimes do prescribe ICI to those patients (1, 2). Although mostly well-tolerated, ICI can cause severe and irreversible immune-related adverse events (irAE), affecting the quality of life and further lines of treatment. Timely recognition of irAE is paramount in order to control them.

We report the case of a patient with advanced NSCLC in whom treatment with pembrolizumab revealed a granulomatosis with polyangiitis (GPA). We then discuss the predictive value of irAE and safety of ICI in patients with PAID.

## Case Presentation

**Figure 1:**
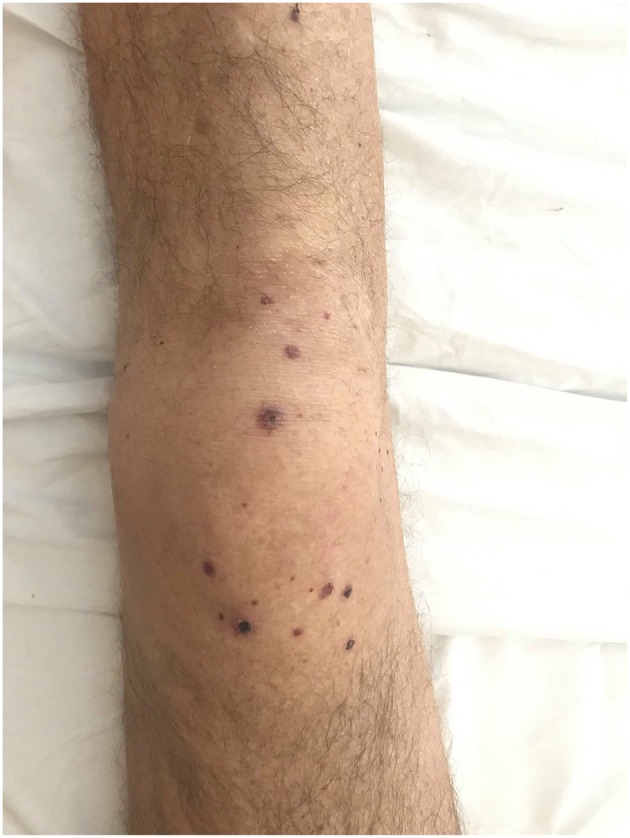
Dark red, necrotic, slightly tender lesions developed symmetrically on MCP joints and knees.

**Figure 2:**
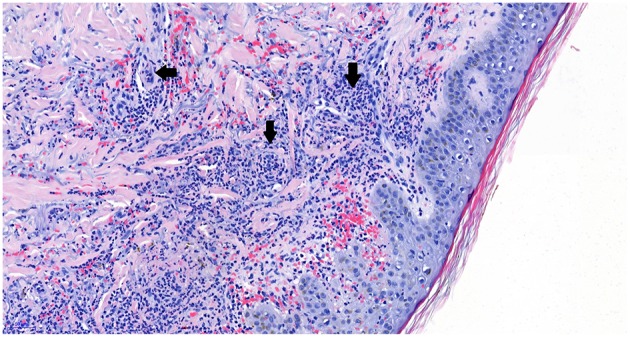
Hematoxylin and eosin (HE) staining shows blood vessels (white areas) with surrounding neutrophilic inflammatory aggregates (arrows), establishing the diagnosis of neutrophilic vasculitis. Picture magnification: 20x; scale bar: 50 μ.

A 64-years old male patient was diagnosed with stage IVB poorly differentiated NSCLC favoring adenocarcinoma of the right upper lobe with several bone lesions (cT4N2M1c). His medical history included a cerebrovascular accident and ischemic heart disease with subacute myocardial infarction in 2003. His chronic medication included acetylsalicylate acid 100 mg once daily (OD) and simvastatin 40 mg 0D, both since 2003. Regarding the tumor no driver mutation was identified by next-generation sequencing analysis. The Programmed Death Ligand-1 (PD-L1) expression level was assessed by immunohistochemistry using a monoclonal antibody to PD-L1 (clone 22C3, Dako) and a Benchmark Ultra (Roche) automated scope with subsequent evaluation by a certified pathologist, revealing 100% staining of a section including at least 100 evaluable tumor cells. Hence, pembrolizumab 200 mg every 3 weeks was started. Ten days after the first dose the patient was admitted to the hospital due to severe myalgia in both lower limbs with severe functional loss. Biochemistry showed creatine kinase (CK) of 1265 IU/L (upper limit of normal (ULN) = 190) and myoglobin of 2361 μg/L (ULN = 72) with normal renal function. Autoimmune serology showed a normal anti-nuclear factor (ANF) titer (1/80) without any characterization (especially for primary immune-mediated myositis with no anti-JO1, PL-7, PL-12, EJ, SRP, Mi-2, MDA-5, HMGCoA reductase) and anti-neutrophil cytoplasmic antibodies (ANCA) with a high titer of anti-PR3 (178 U/mL, ULN = 2); the infectious serology was negative. The statin was taken for several years prior to these symptoms and CK level before the start of the anti-PD-1 was normal. The electroneuromyography before corticoids showed proximal myopathy of moderate intensity without signs of necrosis. The quadriceps biopsy before corticotherapy was normal. He was treated with analgesics, intravenous fluids, and high-dose methylprednisolone (1 mg/kg/day) with favorable evolution. The diagnosis of immune-mediated myositis associated to granulomatosis with polyangiitis (GPA), former Wegener's disease, was established. The anti-PD-1 remained discontinued. Eight months after an initial partial response (PR) to pembrolizumab, progressive disease was noted and second-line doublet chemotherapy was started after antalgic irradiation of a metastatic pelvic mass. Subsequently, PR was noted. A year after the initial presentation of myositis the patient's condition worsened due to dyspnea and arthritis. Evaluation showed a new left-sided pleural effusion and a new lung consolidation. Based on a strong inflammatory syndrome (C-reactive protein (CRP) 116 mg/dL) and a neutrophilic exudate without evidence for empyema the patient was treated with amoxicilline-clavulanate for 14 days. In total, three pleural fluid cultures remained sterile. Due to persistence of the effusion and lack of clinical improvement a pleuroscopy was performed. The fluid appeared unclear and a few non-specific lesions were biopsied on the parietal pleura. They revealed a subacute pleuritis without tumor infiltration, granuloma or vasculitis. The arthritis was symmetrical and located in the wrists, metacarpophalangeal (MCP) joints and knees, without any evidence for infection or crystal-associated disease. A few days later, skin lesions appeared on the MCP and knees (Figure 1). Biopsy there showed a neutrophilic vasculitis, as can be seen in cutaneous forms of GPA (3) (Figure 2). The new lung consolidation was biopsied and showed only necrosis with no specific features of GPA-related lung involvement. Along with this clinical deterioration the autoimmune serology showed a rise in anti-PR3 titer (352.1 U/mL). The CRP dropped dramatically after initiation of corticoids (methylprednisolone at 1 mg/kg/day) along with clear clinical improvement. Recent clinical and radiological evaluation showed that the patient was in good overall condition with no signs of oncological progression despite discontinuation of the chemotherapy. We noted a progression-free survival (PFS) of 10 months after the second line chemotherapy and an overall survival (OS) of 18 months.

## Discussion

With this case we report a unique and rare but severe side effect of pembrolizumab with long-lasting consequences in a NSCLC patient. It remains uncertain whether GPA was preexisting and clinically silent but revealed by the use of the anti-PD-1 or primarily induced by this treatment. Indeed, no autoimmune serology was available from before the start of treatment. Retrospectively, the patient mentioned mild myalgia with normal CK values prior to diagnosis and treatment, responding to corticoids. We therefore think they were possibly signs of a low active GPA.

Immune-related adverse events (irAE) are a kind of adverse events specific to immune checkpoint inhibitors (ICI). Virtually all organs can be affected depicting a wide variety of symptoms. They mostly appear early during treatment but can also appear long after drug discontinuation. Relapses are possible, also in the long term, as seen in our patient (4). Severe irAE occur at a low frequency (~10%) for anti-PD-1/-PD-L1 used in monotherapy (5–9). The general consensus for their management is high-dose corticoids and interruption of the ICI. In the absence of response, adding immunomodulators (mycophenolate mofetyl, anti-TNFα) is recommended (4, 10).

Beside their impact on patients' quality of life and treatment plan, irAE are of specific interest for the clinician. They are considered potential predictors of response to treatment. In NSCLC, several studies showed superior PFS, response rates (RR) and/or OS in patients treated with anti-PD-1/anti-PD-L1 and showing irAE of various types and severity (11–13). The responses were independent of corticoids when needed. These series are, however, small-sized and retrospective. Our patient's tumor showed a PR according to the REsponse Criteria in Solid Tumors (RECIST) version 1.1 with a 50% reduction in the sum of target lesions and a PFS of 8 months after a single dose of pembrolizumab.

Mechanisms of irAE are not yet fully understood. In melanoma, it is argued that there exists a similarity between self-antigens and tumor neoantigens (14). Due to checkpoint inhibition, reactivated and expanding T-cells can interact with both tumor and healthy cells. Preexisting autoimmunity can also explain irAE: preexisting inactivated T-cells directed at self-antigens get reactivated by ICI, clinically translating into irAE (15). ICI might also impact B-cells, either directly via the PD-1 receptor or via the action of T-cells. These effects, however, are not yet fully understood (16).

New onset granulomatous diseases have been described in cancer patients treated with ICI (17, 18). These cases were involving the lungs and/or lymph nodes and pathologically identified as sarcoid-like reactions. To our knowledge, only one case of ICI-induced GPA has been reported in a melanoma patient first treated with ipilimumab, then with pembrolizumab (19). As CD4+ T-cells driven disease, it is understandable that sarcoidosis may be revealed by ICI that will increase the CD4+ T lymphocyte population although the precise mechanisms of this have not been ascertained so far. Wilde et al. suggested the role of the negative coregulatory factor PD-1 in GPA by demonstrating a high level of PD-1 expression in a series of 32 patients as compared to healthy controls and the lack of PD-1 on CD4+ lymphocytes in GPA lesions (20). The IL-17 pathway, known to induce inflammatory and autoimmune phenomena, is also suspected to play a role in GPA as levels of IL-17 producing T cells (Th17) were found to be increased, irrespective of disease activity (21).

Classically, patients known to have an autoimmune disorder have been excluded from clinical trials with ICI. Daily practice, however, shows that this restriction is not always followed. Small-sized studies of such (mainly melanoma) patients treated with an anti-PD-1 are available (1, 2). Reporting on mostly stable PAID, both studies show an increased risk of (new) autoimmune symptoms in ~40% of patients, mainly with low-grade severity. The permanent discontinuation rate due to irAE was 8% (1) vs. 9% (2) which is similar to patients with no PAID (5–9). The type of PAID might also indicate a higher vs. lower risk of flare on anti-PD-1 treatment, as is the case for rheumatologic disorders, closely linked to the PD-1/PD-L1 axis (1). Danlos et al. found an earlier onset of irAE in patients with PAID compared to patients without PAID (2). Both studies found a similar efficacy of ICI in patients with and without PAID on anti-PD-1 in terms of OS and RR. As a consequence, both authors conclude that ICI treatment in patients with PAID is feasible, albeit requiring adequate follow-up and multidisciplinary management. Systematic autoimmune screening before the start of ICI might reinforce the awareness for irAE.

## Conclusion

ICI are commonly used in the treatment of advanced NSCLC. They can, although rarely, induce severe irAE that can cause a major morbidity with potentially long-term effects and that can impact further treatment plan. We describe the first case of GPA in a patient treated with an anti-PD-1 antibody for advanced NSCLC. Although stable PAID does not seem an absolute contraindication to the use of ICI, close monitoring for side effects in these patients seems warranted. Patients presenting an irAE seem to have a favorable oncological outcome. The exact mechanisms of irAE remain unclear.

## Patient Informed Consent

Written informed consent was obtained from the participant for the publication of this case report and any potentially identifying images/information.

## Author Contributions

AS reviewed the literature and wrote the paper. RA and MP followed the patient and collected clinical and biological data. OM analyzed the autoimmune serology data and contributed to the redaction of the manuscript. ND gave the pathology input of this case. RL and BD reviewed and contributed to the final version of the paper. All authors read and approved the final manuscript.

### Conflict of Interest Statement

The authors declare that the research was conducted in the absence of any commercial or financial relationships that could be construed as a potential conflict of interest.
